# Risk factors for children’s blood lead levels in metal mining and smelting communities in Armenia: a cross-sectional study

**DOI:** 10.1186/s12889-016-3613-9

**Published:** 2016-09-07

**Authors:** Ruzanna Grigoryan, Varduhi Petrosyan, Dzovinar Melkom Melkomian, Vahe Khachadourian, Andrew McCartor, Byron Crape

**Affiliations:** 1 School of Public Health, American University of Armenia, 40 Marshal Baghramian Avenue, Yerevan, 0019 Armenia; 2 Department of Epidemiology, School of Public Health, University of California, Los Angeles, Los Angeles, CA USA; 3 Blacksmith Institute for a Pure Earth, New York, USA

**Keywords:** Blood lead level, Children, Smelting, Metal Mining, Lead exposure, Lead contamination

## Abstract

**Background:**

Children’s exposure to lead poses a significant risk for neurobehavioral consequences. Existing studies documented lead contamination in residential soil in mining and smelting communities in Armenia. This study aimed to assess blood lead levels (BLL) in children living in three communities in Armenia adjacent to metal mining and smelting industries, and related risk factors.

**Methods:**

This cross-sectional study included 159 children born from 2007 to 2009 and living in Alaverdi and Akhtala communities and Erebuni district in Yerevan - the capital city. The BLL was measured with a portable LeadCare II Blood Lead Analyzer; a survey was conducted with primary caregivers.

**Results:**

Overall Geometric Mean (GM) of BLL was 6.0 μg/dl: 6.8 for Akhtala, 6.4 for Alaverdi and 5.1 for Yerevan. In the sample 68.6 % of children had BLL above CDC defined reference level of 5 μg/dl: 83.8 % in Akhtala, 72.5 % in Alaverdi, and 52.8 % in Yerevan. Caregiver’s lower education, dusting furniture less than daily, and housing distance from toxic source(s) were risk factors for higher BLL. Additional analysis for separate communities demonstrated interaction between housing distance from toxic source(s) and type of window in Erebuni district of Yerevan.

**Conclusions:**

The study demonstrated that children in three communities adjacent to metal mining and smelting industries were exposed to lead. Investigation of the risk factors suggested that in addition to promoting safe industrial practices at the national level, community-specific interventions could be implemented in low- and middle-income countries to reduce BLL among children.

## Background

Lead is a toxic heavy metal that poses significant harm to human health, especially to children [1]. The most evident health damage of lead exposure in children is decreased intelligence quotient and neurobehavioral development [2]. The active hand-to-mouth behavior of children contributes to increased risk of lead exposure [3]. Children are more vulnerable to lead exposure because their digestive tract absorbs up to 50 % of the lead ingested (compared to 10–15 % in adults), the dose of lead contamination per unit body weight is higher and their developing brains are more susceptible to lead compared to adults [4]. There is no safe level of lead for children; even low lead levels in blood can significantly affect children’s cognitive abilities [5]. The US Centers for Disease Control and Prevention (CDC) recommends 5 μg/dl reference level of lead in the blood of children from 1 to 5 years old. This reference level presents 97.5 percentile of blood lead data distribution in 1–5 years old children in the US, which means that 97.5 % of children of this age in the US have BLL below 5 μg/dl [6].

Socio-demographic and behavioral characteristics associated with higher blood lead levels (BLL) among children include: male gender, younger age, longer outdoor hours, not washing hands before eating, caregiver’s lower education and family’s low income [3, 7–13]. Environmental risk factors associated with higher BLL include lead levels in residential soil and house dust, living in lead emitting industrial areas, a parent employed in lead industry, and exposure to secondhand smoke [3, 7, 10, 14, 15].

Lead exposure is a serious problem for low- and middle-income countries where lead emitting industries are often less tightly regulated than in high-income countries [16]. The Republic of Armenia (Armenia) is a lower-middle-income country [17, 18]. In Armenia, metal mining and smelting industries are the main sources of lead pollution [19]. Mining and quarrying is the largest producing industry in the country by volume of industrial production and accounts for half of the country’s exports [17, 20]. There are 670 mines in the country, including 22 active base metal mines and 19 tailing ponds [20, 21]. The use of lead-based paint was banned in 1920 (International Labor Office 1927), and the use of leaded gasoline was largely phased out in 1998 (Kurkjian et al., 2002) and banned in 2000 (RA Government, 2000) [22–24].

Alaverdi and Akhtala are towns in the northern Lori province (marz) of Armenia with populations of 16,400 and 2400, respectively [25]. There is a copper smelter in Alaverdi with an annual capacity of processing up to 50,000 tons of copper concentrate and 10,000 tons of blister copper [26].

Akhtala has a processing facility and an open pit barite-poly-metallic mine with an annual production capacity of 12,000 tons of copper concentrate [27]. The mine has three tailing ponds, one of which is located in the center of the town and is currently non-operational [21].

Yerevan is the capital city of Armenia with a population of 1,054,698 people [28]. There are two smelters located in Yerevan’s Erebuni district. One of the smelters processes molybdenum concentrates and produces metal alloys [29]. The other smelter produces firon-molybdenum alloy with 3600 tons of annual production [30].

Petrosyan et al. [19] demonstrated that Alaverdi and Akhtala towns had significant lead contamination in residential soil in 2001. A recent detailed ecological risk assessment found that 24.0 % of soil samples in Alaverdi and 27.1 % in Akhtala exceeded the US Environmental Protection Agency hazard standard of lead in bare soil in play areas of 400 mg/kg [31, 32].

Despite documentation of lead contamination in Alaverdi and Akhtala, the impact of the mining and smelting industries on public health has not been previously assessed. The present study aimed to a) measure the BLL in children from Alaverdi, Akhtala and Erebuni district of Yerevan, b) assess the potential risk factors for elevated BLL in those communities and check for interactions between the risk factors.

## Methods

### Study aim, design and population

A cross-sectional study was designed to achieve the study objectives. Children born in 2007, 2008 and 2009 residing in Alaverdi, Akhtala and Yerevan’s Erebuni district whose main caregivers were available for face-to-face interviews at the moment of taking blood samples were eligible for the study. To minimize the possibility of lead exposure outside the community, the following exclusion criteria were set: a) children who had been absent from their residential area longer than 10 days during the last month, and b) children who had been absent from their residential area longer than 3 months during the last year. Selection and recruitment of children was conducted through local medical registries of the local healthcare facilities.

The study team took the sampling frames from the local primary healthcare facilities. All 46 eligible children registered in the primary healthcare center of Akhtala were invited to participate in the study. The research team selected 94 children born from 2007 to 2009 living in Alaverdi through simple random sampling from the registration lists of the primary healthcare polyclinic comprising about 21 % of all 441 children born during 2007–2009. Three polyclinics in Yerevan Erebuni district serve children living in the community. Overall, 86 children born during 2007–2009 were selected through a multistage, probability-proportional-to-size cluster sampling technique proportionate to the size of the population served by each polyclinic. The sample comprised about 3 % of all 3239 eligible children in Yerevan Erebuni district.

The trained interviewers asked the child’s main caregiver to respond to a questionnaire, which included a set of questions on family’s socio-demographics characteristics, housing conditions - such as cleaning practices and exposure to second hand smoke -, caregivers’ knowledge about lead exposure and preventive measures, child’s health, hygiene and nutrition, and potential routes for both indoor and outdoor lead exposure. Following the BLL testing and the interview with caregivers, the researchers informed caregivers about their child’s BLL results, counseled on evidence-based preventive measures to minimize lead exposure and provided with an information brochure. The field work took place during fall of 2013.

### BLL and anthropometric measurements

All the children underwent capillary blood lead, and height and weight measurements. A trained pediatric nurse collected all the blood samples following the US Center for Disease Control and Prevention (CDC) recommended finger stick method and analyzed them for BLL using the portable LeadCare II Blood Lead Analyzer with a detection range of 3.3–65.0 μg/dl [33, 34]. The LeadCare II Point-of-Care (POC) device was developed in collaboration with the CDC. The Food and Drug Administration (FDA) has classified this device as CLIA-waived (Clinical Laboratory Improvement Amendments) and approved for use at non-traditional laboratory settings. The results of the CLIA waiver clinical field trials, which compared the tests results obtained through this device to those obtained on graphite furnace atomic absorption spectrometry (GFAAS), revealed a 0.979 overall correlation [35, 36]. We carefully followed the quality control procedures recommended by the manufacturer [34].

### Variables and statistical analysis

We conducted double entry of collected data, cleaned and analyzed the dataset with the SPSS 16.0. The outcome variable was the continuous variable of child BLL. There were three children in the sample, who had blood lead levels below the detection level of the LeadCare II Analyzer, which is 3.3 μg/dl. For these cases the Analyzer provided the estimated range of the measurement (0 to 3.3 μg/dl), instead of a specific level. Thus, the BLL measurements of these three children were replaced with the midpoint of the range (1.6 μg/dl). To meet the normality assumption of the linear regression we made a natural log transformation of the positively skewed BLL variable. We calculated child’s nutrition % score based on the frequency of having lead-preventive food items, such as milk, yogurt, cheese, beans, meat, dry fruits, dark chocolate, seeds, and dark green-leaved vegetables. The higher the child’s nutrition % score the more often the child had those foods in his/her diet.

The caregiver’s knowledge % score was calculated based on the correct answers to the 28 questions measuring caregivers’ knowledge on how to prevent lead exposure in children. The higher % score is an indicator of caregiver’s higher knowledge.

The household living standard % score was calculated based on three questions: employment rate (% of employed adult family members), caregiver’s perceived standard of living, and family’s average monthly spending. The higher % score shows that the family had a higher prosperity level.

The household hygiene % score was calculated based on the cleaning and hygiene practices in the house, such as dry vacuum or wet cleaning of floor surfaces, dusting furniture surfaces, cleaning the soles of shoes and taking them off when entering the house. The higher the household hygiene % score, the more preventive measures were taken in the house to reduce the level of lead exposure due to outdoor lead pollution.

The child’s hygiene score was based on the frequency with which the child washed his/her hands after returning home from the yard, before having a meal, as well as based on whether or not they had a habit of biting their nails.

**Fig. 1:**
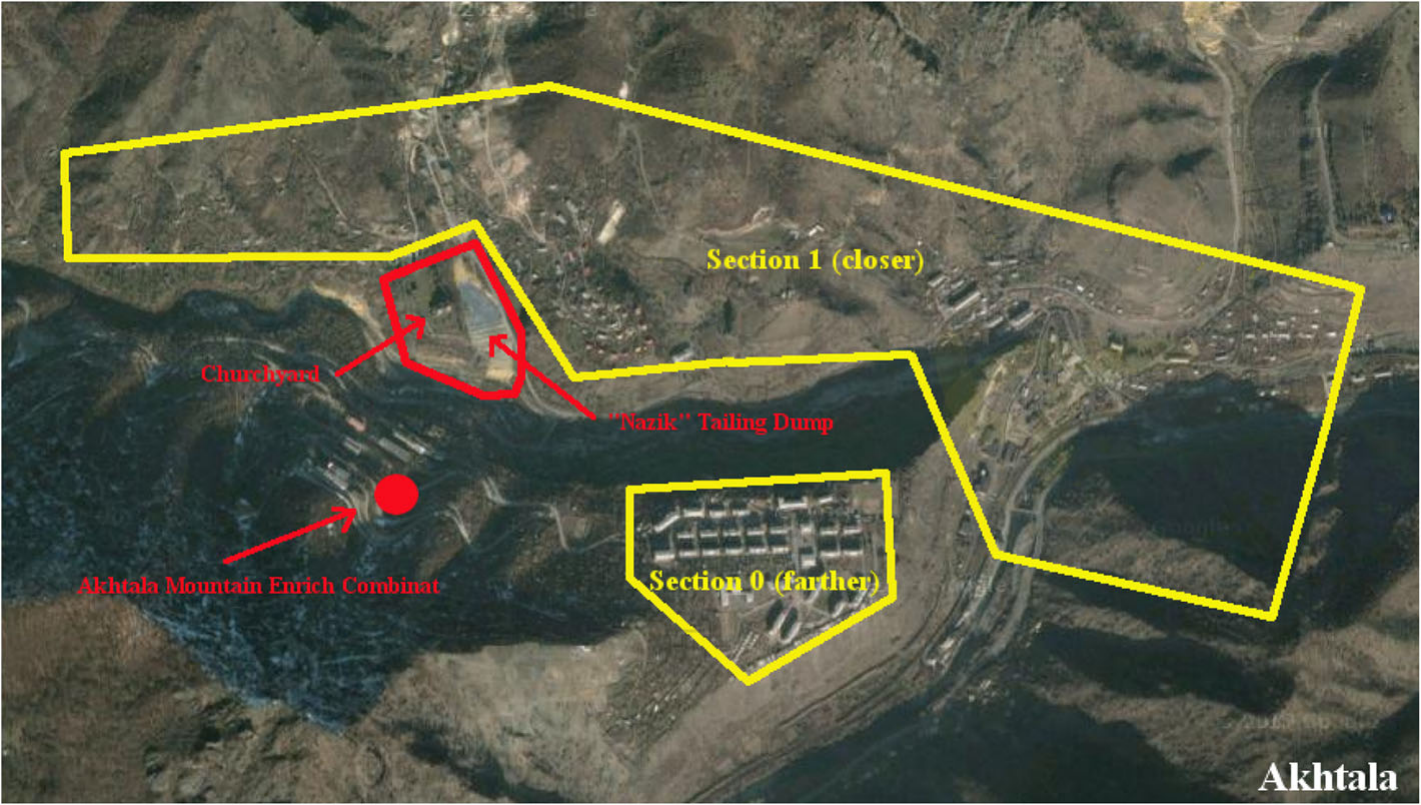
Akhtala Community. The farther section was considered to have less exposure because apart from being located slightly farther from the toxic sources it was also located above the gorge on top of the hill as opposed to the closer section which was in the gorge

**Fig. 2:**
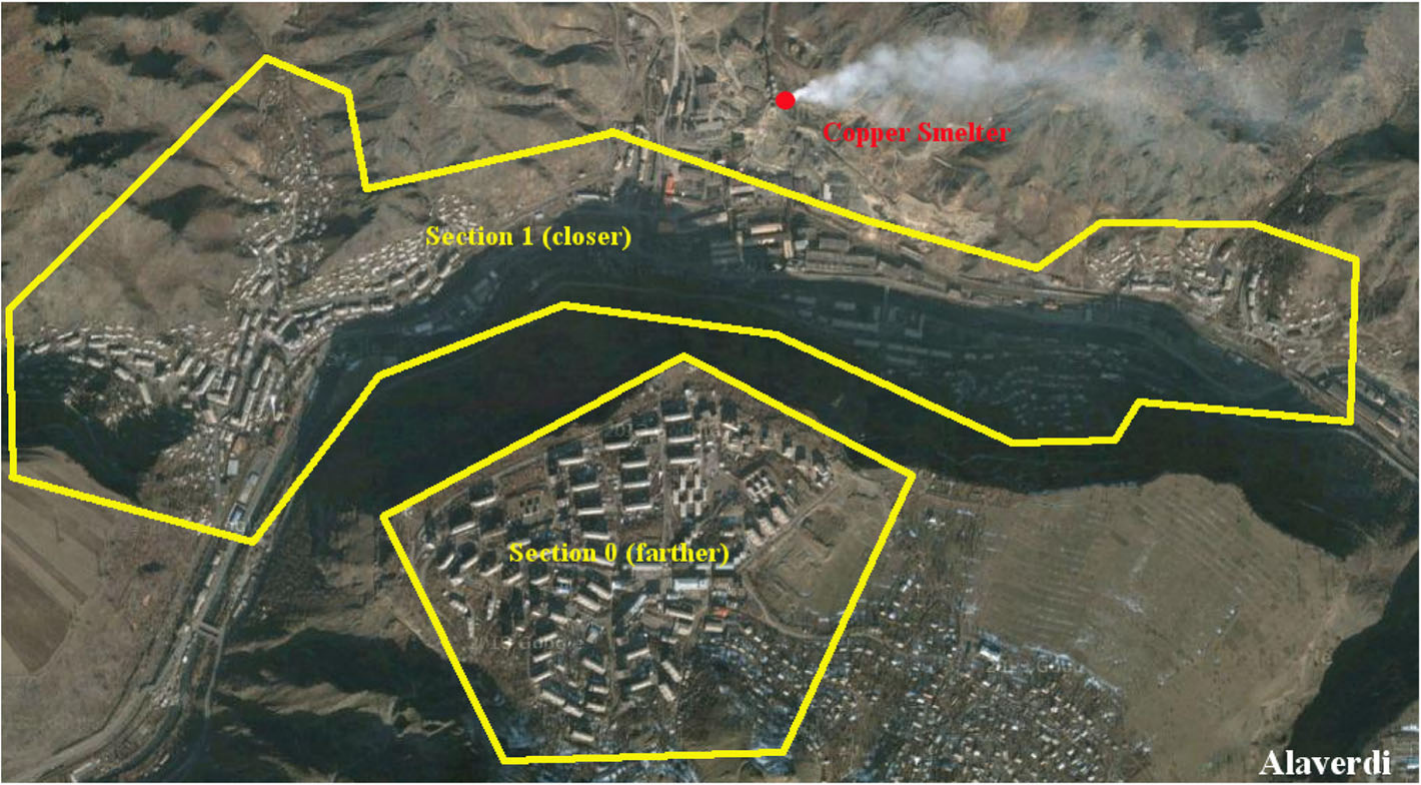
Alaverdi Community. The farther section was considered to have less exposure because apart from being located slightly farther from the toxic sources it was also located above the gorge on top of the hill as opposed to the closer section which was in the gorge

**Fig. 3:**
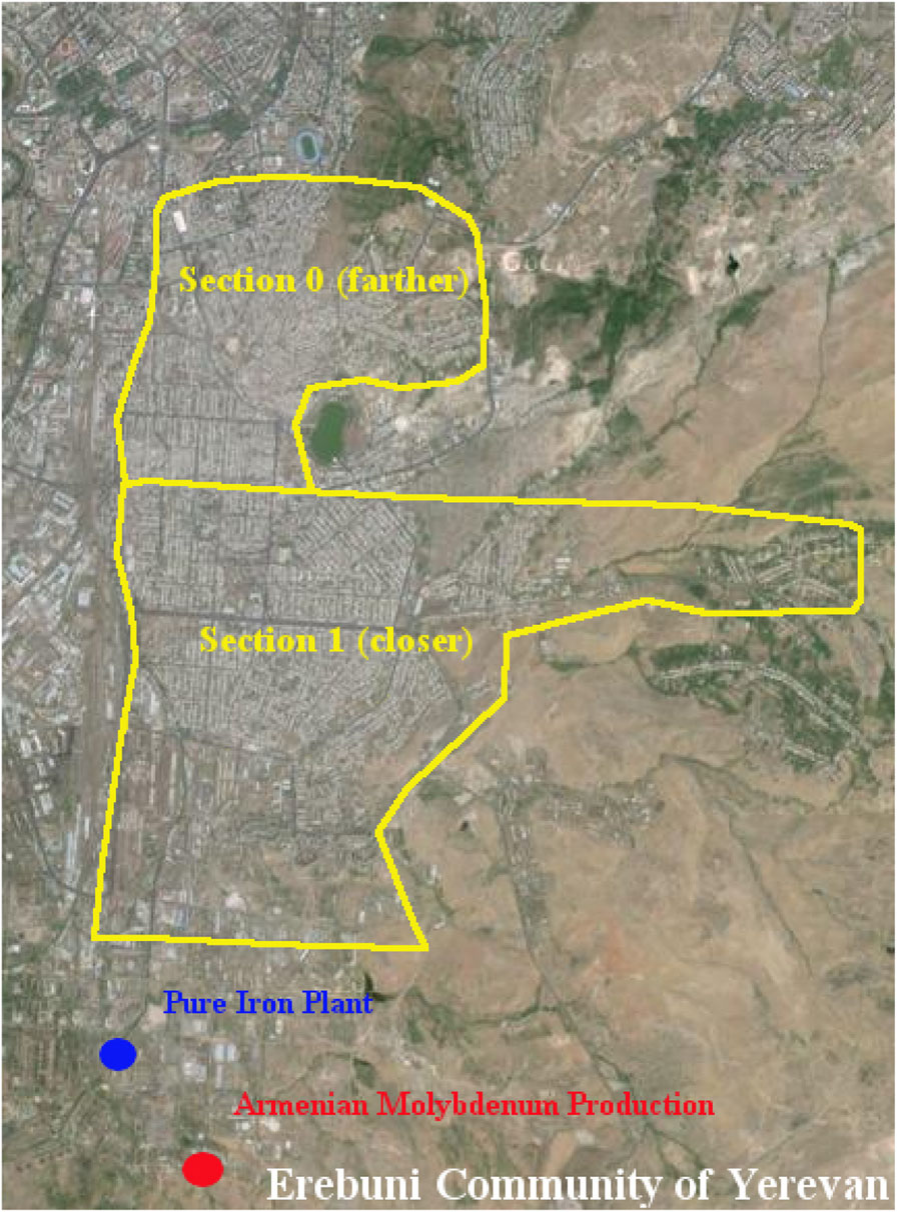
Erebuni District of Yerevan

The communities were divided into two sections – those located closer to the toxic source and those located farther from the toxic source. In Akhtala and Alaverdi those districts that were located in the gorge were defined as closer sections, while the districts above the gorge on top of the hill were defined as farther. In Yerevan this division was based on the geographical distance from the toxic source and was derived from official administrative subdivisions of Erebuni district of Yerevan. Figures 1, 2, and 3 present the locations of the toxic source(s) and depict the areas closer and farther from the toxic source(s) in Akhtala, Alaverdi, and Yerevan, respectively. We calculated the midpoint distance from the toxic source variable via Google map. It shows the distance of the midpoint of the closer and farther sections of the community from the toxic source (or from the midpoint of toxic sources, if there was more than one toxic source in a certain community).

The stunting (low height for age) variable was based on the WHO definition as an anthropometric value below 2 standard deviation or Z-scores less than 2.3rd percentile [37]. We categorized the variable distance of child’s housing from the toxic source(s) for each community into closer and farther. The type of windows was a binary variable: new and old. New windows were defined as double glazed and vacuum sealed windows. Old windows stands for all other types, including those that were installed during the Soviet times or had been partially replaced with new ones. Caregiver’s education was also a binary variable: higher education meant that the caregiver had more than 13 years of education - including years spent in primary and secondary schools and lower education meant 13 or less years of education.

The research team checked the Loess curve and cell sizes to explore data distribution and to categorize data assuring adequate cell size. To assess the associations between each independent variable and the outcome variable of natural log transformed BLL unadjusted linear regressions were conducted. The research team checked the assumptions of linearity, normality and equal variability of residuals for linear regressions. We created dummy variables to compare the groups with the reference group for variables with more than two categories. The exponentiated coefficients of natural log transformed BLL were interpreted as ratios of geometric mean (GM) of BLL between the comparison and reference group. Those independent variables that were at least marginally statistically significantly associated with the outcome variable in the unadjusted linear regression analysis (p ≤ 0.1) were included in the multivariable linear regression (MLR) analysis [38]. The model with the highest R^2^ was selected as the final one. We checked for collinearity between the covariates by examining tolerance, variance inflation factor and correlations between the variables. We assessed the interactions between covariates by introducing interaction terms. The unadjusted and multivariable regression analyses were conducted for the total sample and then for the sample from Yerevan and that of Akhtala and Alaverdi combined. The two communities were combined with the consideration of increasing the power of the sample and to be able to demonstrate true associations. Moreover, they were not statistically significantly different from each other in terms of the outcome variable of BLL.

## Results

The research team recruited 159 eligible children in October-November 2013: 37 from Akhtala, 69 from Alaverdi and 53 from Yerevan. The response rate for the entire sample was 70.4 %. Response rate per town was: 80.4 % in Akhtala, 73.4 % in Alaverdi and 61.6 % in Yerevan. Among the main caregivers 90.6 % were mothers, 8.2 % grandmothers, 0.6 % fathers and 0.6 % grandmother’s sister. The majority of respondents (caregivers) were women (99.4 %). The mean age ± SD was 32.3 ± 8.1 years old. The majority of respondents (96.2 %) were married and the remaining (3.8 %) were widowed. Almost one quarter (25.8 %) had more than 13 years of education, the rest had lower. More than three quarters of the respondents (76.1 %) were unemployed.

Gender distribution among children was 52.5 % boys and 47.5 % girls. The mean ± SD age of children was 5.3 ± 0.9. In the total sample, 68.6 % of children had BLL above CDC defined reference level of 5 μg/dl. The percentage of children exceeding the reference level was statistically significantly different between communities (*p* = 0.005): 83.8 % of children in Akhtala, 72.5 % in Alaverdi, and 52.8 % in Yerevan. The geometric mean (GM) of BLL was 6.0 ± 1.5 μg/dl. The GMs of BLL were also statistically significantly different in three communities (*p* = 0.001): 6.8 μg/dl in Akhtala, 6.4 μg/dl in Alaverdi and 5.1 μg/dl in Yerevan. Table 1 presents the descriptive statistics for the total sample and for each community.

**Table 1:** Descriptive statistics by communities and for the total sample, 2013

Variables	%, mean, range, SD	Alaverdi (SS^a^ = 69)	Akhtala (SS = 37)	Yerevan (SS = 53)	Total (SS = 159)
Children involved in the study	%	43.4	23.3	33.3	100.0
BLL above 5 μg/dl	%	72.5	83.8	52.8	68.6
GM of BLL	Mean ± SD	6.4 ± 3.1	6.8 ± 3.2	5.1 ± 2.4	6.0 ± 3.0
	Range	3.5–24.0	3.6–15.5	1.6–11.7	1.6–24.0
Caregivers’ age	Mean ± SD	31.8 ± 8.43	32.14 ± 7.23	33.06 ± 8.17	32.3 ± 8.1
Caregiver’s education: higher versus lower	Higher %	18.8	24.3	35.85	25.8
Caregivers’ marital status: married versus widowed	Married %	97.1	94.6	96.2	96.2
Caregivers’ employment status	Employed %	21.7	10.8	35.6	23.9
Children’s sex	Female %	46.4	48.6	48.1	47.5
Child’s age in years	Mean ± SD	5.2 ± 0.76	5.51 ± 0.83	5.35 ± 0.99	5.3 ± 0.9
Child nutrition % score	Mean ± SD	50.96 ± 15.64	49.22 ± 13.75	49.53 ± 15.56	50.0 ± 15.1
Stunting in children	Yes %	9.4	19.4	10.6	12.2
Child plays with soil in yards, play grounds or gardens in warm seasons	Yes %	79.1	75.7	69.2	75.0
Hours spent in yards, playgrounds or gardens daily in warm season	Mean ± SD	3.3 ± 2.8	4.1 ± 2.9	2.6 ± 2.8	3.3 ± 2.8
Frequency of child washing hands after coming home: always versus not always	Always %	89.9	66.7	90.4	84.7
Frequency of child washing hands before eating: always versus not always	Always %	67.6	56.8	63.5	63.7
Child’s behavior of biting nails	Yes %	17.4	21.6	13.5	17.1
Child’s hygiene score	Mean ± SD	1.74 ± 0.68	1.44 ± 0.84	1.67 ± 0.59	1.7 ± 0.7
Household living standard % score	Mean ± SD	35.9 ± 13.3	30.4	40.7 ± 14.3	36.3 ± 14.3
Household size	Mean ± SD	4.97 ± 1.14	5.03 ± 1.46	5.51 ± 1.40	5.2 ± 1.3
Housing type^b^: flat or house	Flat %	89.9	70.3	45.3	70.4
Housing floor^b^: first versus higher floor	First floor %	34.8	56.8	58.5	47.8
Type of windows: new versus old	New %	13.0	18.9	41.5	23.9
Daily mean duration of opening the windows in summer -in hours	Mean ± SD	14.80 ± 6.11	17.76 ± 7.35	19.12 ± 7.50	16.9 ± 7.1
Having carpet on the floor	Always %	56.5	54.1	50.9	54.1
	Seasonal %	34.8	29.7	34	33.3
	Never %	8.7	16.2	15.1	12.6
Duration of occupying the current flat/house in years	Mean ± SD	8.7 ± 8.6	7.9 ± 7.4	9.5 ± 8.5	8.8 ± 8.3
Number of current smokers in the family	Mean ± SD	1.1 ± 0.6	0.9 ± 0.6	1.3 ± 0.8	1.1 ± 0.7
Smoking in the presence of the child	Yes %	55.9	85.7	76.1	69.2
Having a family member working in a processing facility, mine or smelter compared to not having any	Yes %	29.0	58.3	0	25.9
Number of family members working in a processing facility, mine or smelter	Mean ± SD	0.4 ± 0.6	0.6 ± 0.6	0 ± 0	0.3 ± 0.5
Caregiver’s knowledge % score	Mean ± SD	53.6 ± 17.7	58.8 ± 19.1	42.2 ± 18.1	52.3 ± 18.6
Frequency of parents changing clothes/shoes before coming from processing facility, mine or smelter	Always %	85.0	71.4	100	78.0
	Not always %	15.0	28.6	0	22.0
Household hygiene % score	Mean ± SD	67.4 ± 13.1	68.4 ± 13.7	74.6 ± 12.9	70.4 ± 13.5
Frequency of furniture dusting^c^: daily versus less than daily	Daily %	92.6	83.8	98.1	92.4
Housing distance from the toxic source(s)	Closer %	59.4	56.8	64.2	60.4
	Farther %	40.6	43.2	35.8	39.6
Midpoint distance from the toxic source	Closer	640	846	3 516	
(meters)	Farther	1 318^d^	849^d^	5 493	

^a^ Sample size

^b^ House is defined as a stand-alone building that consists of one or two floors. A total of 12 out of 47 houses had two floors. Flats are apartments in multi-floor buildings. When calculating the variable of living on the first floor or higher floors the houses with second floor were included in the category of first floor

^c^ When the individual questions of the Household protective hygiene % score were analyzed only the variable of dusting furniture was statistically significantly associated with BLL. Therefore, it is presented in descriptive statistics and regression analysis

^d^ This section was considered to have less exposure because apart from being located slightly farther from the toxic sources it was also located above the gorge on top of the hill as opposed to the closer section which was in the gorge

The final MLR model included the following variables: town, caregiver’s education, dusting furniture, and distance from the toxic source(s) (Table 2). Caregiver’s lower education compared to higher education was associated with 23 % higher GM of BLL adjusted for other variables (CI = 1.07, 1.41, *p* = 0.004). Dusting furniture less than daily compared with daily dusting was associated with 29 % higher GM of BLL adjusted for other variables (CI = 0.98, 1.55, *p* = 0.075). Living in Akhtala or Alaverdi compared to Yerevan was associated with 24 % higher GM of BLL adjusted for other variables (CI = 1.09, 1.42, *p* = 0.001). Closer housing distance from the toxic source(s) compared to farther housing distance was associated with 22 % higher GM of BLL adjusted for other variables (CI = 1.08, 1.38, *p* = 0.002).

**Table 2:** Final multivariable linear regression models for the total sample, for the sample from Yerevan and for the combined sample from Akhtala and Alaverdi

Variables	Adjusted ratio of expected GM of BLL (95 % CI)	*p* value
Total sample		
Combined Akhtala and Alaverdi compared to Yerevan	1.24 (1.09, 1.42)	0.001*
Caregiver’s lower education compared to higher	1.23 (1.07, 1.41)	0.004*
Dusting furniture less often than daily compared to daily	1.29 (0.98, 1.55)	0.075**
Closer housing distance from toxic source(s) compared to farther	1.22 (1.08,1.38)	0.002*
Akhtala and Alaverdi combined		
Caregiver’s lower education compared to higher	1.20 (1.01, 1.43)	0.035*
Dusting furniture less often than daily compared to daily	1.30 (1.04, 1.64)	0.024*
Yerevan		
Caregiver’s lower education compared to higher	1.28 (1.02, 1.59)	0.030*
For housing located farther from toxic source(s)***		
Old windows compared to new ones	2.01 (1.42, 2.86)	0.000*
For housing located closer to the toxic source(s)		
Old windows compared to new ones	1.06 (0.82, 1.37)	0.628

* Statistical significance (p ≤ 0.05)

** Marginally statistical significance (0.05 > p ≤ 0.1)

*** Interaction term between housing distance from toxic source(s) and type of window was statistically significant (*p* < 0.004)

### Results for the combined sample from Akhtala and Alaverdi

Caregiver’s lower education compared to higher education was statistically significantly associated with 20 % higher GM of BLL adjusted for the other variable (CI = 1.01, 1.43, *p* = 0.035). Dusting furniture less than daily compared to daily was associated with 30 % higher GM of BLL, adjusted for the other variable (CI = 1.04, 1.64, *p* = 0.024).

### Results for the sample from Yerevan

The MLR revealed that caregiver’s education, type of windows, housing distance and interaction between residential distance and types of windows were associated with GM BLL (Table 2). Caregiver’s lower education compared to higher education was associated with 28 % higher GM of BLL adjusted for other variables (CI = 1.02, 1.59, *p* = 0.030).

After adjusting for caregiver’s education, only for those who lived at closer distance from the toxic source(s) the type of windows was not statistically significantly associated with the GM of BLL (CI = 0.82, 1.37, *p* = 0.628). For those who lived at farther distance from the toxic source(s) having old windows compared to new windows was statistically significantly associated with a 101 % increase of the GM of BLL (CI = 1.42, 2.86, *p* = 0.000).

## Discussion

Our study assessed BLL of 3.9–6.9 years old children in three communities of Armenia– Akhtala, Alaverdi and Yerevan - each with different levels of residential soil lead contamination and different sources of contamination. In our sample from three communities BLL of 68.6 % of children exceeded the CDC defined reference level of 5 μg/dl. This finding indicates that children living in those three communities in Armenia have much higher exposure to lead than children in the US where only 2.5 % of children have BLL above 5 μg/dl [6]. According to our study the most vulnerable communities are Akhtala and Alaverdi followed by Erebuni district of Yerevan. The level of children’s exposure to lead was the highest in Akhtala and the lowest in Yerevan. Children in Alaverdi were more similar to children in Akhtala in terms of lead exposure measure by BLL. This degree of lead exposure in three communities was consistent with findings of an ecological risk assessment that investigated lead levels in residential soil samples. This ecological risk assessment found that 27.1 % of soil samples in Akhtala (GM 307.8 mg/kg), 24.0 % in Alaverdi (GM 234.9 mg/kg), and none in Yerevan (GM 48.3 mg/kg) exceeded the US Environmental Protection Agency’s hazard standard for lead in bare soil in play areas of 400 mg/kg [31].

Our findings indicate that children in the selected communities of Armenia are exposed to lead which according to the existing literature is associated with health hazards including impaired neurobehavioral development, decreased intelligence quotient, poor memory, attention deficit/hyperactivity disorder and decreased growth [2, 39–42]. These risks are especially higher in Akhtala and Alaverdi where both the percentage of children having BLL above the reference level and the geometric mean of BLL were remarkably higher than the same measurements for Yerevan. In Akhtala and Alaverdi the percentage of children above the reference level of 5 μg/dl is almost 34 and 29 times, respectively, higher than the percentage of children with BLL above the reference level in the US.

Similar to other studies, our findings indicated that caregiver’s lower education was a risk factor for child’s higher BLL [3, 13]. Our study also suggested that dusting furniture less than daily was a risk factor, consistent with studies that demonstrated associations between floor dust lead loading and higher BLL [3, 43]. Initial site screenings in polluted communities in Armenia suggested that mining communities in Armenia face the issue of heavy exposure to industrial dust due to open pit mining and explosions near residential areas; moreover, open trucks with mining ore and waste regularly drive through communities resulting in more dust-related pollution [31, 44]. The metal smelting communities face heavy exposure to smelter emissions rich in heavy metals and sulfur dioxide. The community use of slag from the smelter which contains high levels of lead and other heavy metals serve as an additional source of exposure to heavy metals [31, 44].

Living in close proximity to the contamination source(s) compared to living farther away was associated with higher BLL. Similar findings were reported by Boseila et al. [45], who showed that closer distance to the smelter was associated with higher levels of BLL.

The separate analyses for children living in Erebuni district of Yerevan demonstrated that at farther distance from the pollution source(s) the new windows could serve as potential means of protection against smelting related pollution by heavy metals. This could potentially be explained by the fact that at closer distances where the contamination level is usually higher, lead exposure might be through pathways other than the windows. This finding brings a unique insight for further investigation of protective effects of double glazed and vacuum sealed windows in smelting and mining communities not only in Armenia but elsewhere.

Due to limited resources we were not able to measure the lead concentrations in the residential soil for each child to investigate the specific associations between residential soil lead contamination and the BLL. We did not have data to draw conclusions about water source pollution and its potential contribution to elevated BLL in children. The smaller sample size especially in separate communities might have limited the power of the study to find true associations between BLL and other risk factors; to address this issue we combined data from Akhtala and Alaverdi communities and analyzed them together.

## Conclusions

Our findings demonstrated that children in three communities in Armenia adjacent to metal mining and smelting industries were exposed to lead. The results of the study suggested that in parallel to the urgent need for promoting and enforcing safe industrial practices and tighter environmental regulations in the country, community-specific interventions focusing on factors that had protective effect on children’s BLL could be implemented in Armenia and other low- and middle-income countries with metal mining and smelting industry to reduce lead exposure and related health issues among children.

Future research including assessment of neurobehavioral consequences of elevated BLL among exposed children in Armenia would help to convince the policymakers to take serious actions to protect the health of community members, including children. Similar studies in other mining and smelting communities could help to develop affordable recommendations to help those communities to reduce the potential for lead and other heavy metal exposure.

## Abbreviations

BLL: Blood lead level
CDC: Centers for Disease Control and Prevention
GM: Geometric mean
SPSS 16.0: Statistical package for the social sciences 16.0
MLR: Multivariate linear regression
SD: Standard deviation
